# Identification of expression QTL (eQTL) of genes expressed in porcine M. longissimus dorsi and associated with meat quality traits

**DOI:** 10.1186/1471-2164-11-572

**Published:** 2010-10-16

**Authors:** Siriluck Ponsuksili, Eduard Murani, Manfred Schwerin, Karl Schellander, Klaus Wimmers

**Affiliations:** 1 Leibniz Institute for Farm Animal Biology, Research Group Functional Genome Analysis, Wilhelm-Stahl-Allee 2, 18196 Dummerstorf, Germany; 2 Leibniz Institute for Farm Animal Biology, Research Unit Molecular Biology, Wilhelm-Stahl-Allee 2, 18196 Dummerstorf, Germany; 3 Institute of Animal Science, Animal Breeding and Husbandry Group, University of Bonn, Endenicher Allee 15, 53115 Bonn, Germany

## Abstract

**Background:**

Genetic analysis of transcriptional profiles is a promising approach for identifying and dissecting the genetics of complex traits like meat performance. Accordingly, expression levels obtained by microarray analysis were taken as phenotypes in a linkage analysis to map eQTL. Moreover, expression levels were correlated with traits related to meat quality and principle components with high loadings of these traits. By using an up-to-date annotation and localization of the respective probe-sets, the integration of eQTL mapping data and information of trait correlated expression finally served to point to candidate genes for meat quality traits.

**Results:**

Genome-wide transcriptional profiles of *M. longissimus dorsi *RNAs samples of 74 F2 animals of a pig resource population revealed 11,457 probe-sets representing genes expressed in the muscle. Linkage analysis of expression levels of these probe-sets provided 9,180 eQTL at the suggestive significance threshold of LOD > 2. We mapped 653 eQTL on the same chromosome as the corresponding gene and these were designated as 'putative *cis-*eQTL'. In order to link eQTL to the traits of interest, probe-sets were addressed with relative transcript abundances that showed correlation with meat quality traits at p ≤ 0.05. Out of the 653 'putative *cis-*eQTL', 262 transcripts were correlated with at least one meat quality trait. Furthermore, association of expression levels with composite traits with high loadings for meat quality traits generated by principle component analysis were taken into account leading to a list of 85 genes exhibiting *cis-*eQTL and trait dependent expression.

**Conclusion:**

Holistic expression profiling was integrated with QTL analysis for meat quality traits. Correlations between transcript abundance and meat quality traits, combined with genetic positional information of eQTL allowed us to prioritise candidate genes for further study.

## Background

Genetical genomics as a new approach which combines gene-expression data and marker genotypes in a segregating population, offers great perspectives to make a major contribution to the dissection of complex traits [1,2]. Genetical genomics aims at detecting genomic loci that control variation in gene expression, so-called expression QTL (eQTL; to distinguish them from functional QTL that affect traits at the whole-organism level, subsequently termed pheneQTL(pQTL)). The detected eQTL can represent a locus that lies close to the gene that is being controlled (*cis*-acting) or one or more loci that are unlinked to the gene that is being controlled (*trans*-acting) [1]. Expression-QTL for genes showing high correlation with the phenotype may provide the necessary information required for identifying genes that control quantitative phenotypes. Those *cis*-eQTLs resulting from the correlation of expression profiles with phenotypic measurements represent candidate genes for the genetic regulation underlying the variation of the physiological traits [3,4].

We have previously reported on the identification of eQTL of genes showing expression levels correlated with waterholding capacity of meat measured as drip loss [5]. Here in this study, we expand and up-dated the analysis towards global eQTL mapping of transcripts showing variable abundance in porcine muscle and using annotation and localisation data of Affymetrix microarray probe-sets based on the current porcine sequence information [6]. We focus on eQTL of genes whose expression at slaughter is significantly correlated to technological meat quality traits addressed in commercial pig breeding schemes. Taking into account own observations on the reliability of eQTL mapping we highlight eQTL located on the same chromosome as the corresponding genes themselves [7].

## Results

### Summary analysis of eQTL detection

**Figure 1:**
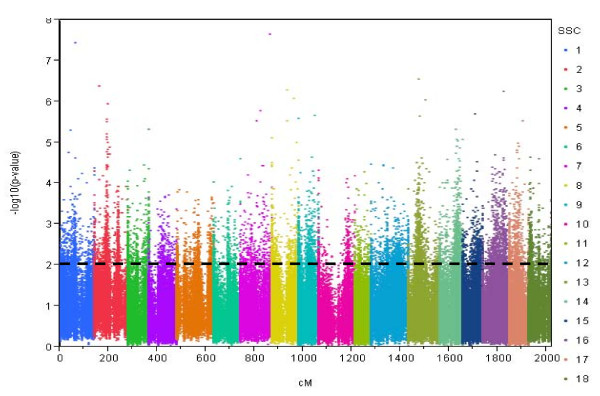
**Graphical scheme of eQTL across 18 chromosome obtained by eQTL analysis of 11457 probe-set.** Y-axis shows P-values, expressed on the -log10 scale, and X-axis shows position of eQTL in cM though18 chromosome. The genome-wide significant threshold (p ≤ 0.05) is shown with the dot line.

Expression data were obtained from *M. longissimus dorsi *samples of 74 F2 animals of a resource population using Affymetrix Porcine Genome Arrays containing in total 24,123 probe-sets of which 20,689 probe-sets were assigned to a known gene [6]. MAS5 analysis revealed consistent 'present calls' for 11,457 probe-sets. This pre-selected set was further analyzed with the more sophisticated hybrid algorithm PLIER [8,9] and the expression levels were subjected to linkage analysis. Out of 11,457 probe-sets 6,117 showed at least one eQTL at the 5% chromosome-wide significance threshold (average F-value 4.92, corresponding to LOD score = 2.0 and nominal significance of p ≤ 10^-3^) corresponding approximately to the suggestive linkage threshold proposed by Lander & Kruglyak [10] (Table 1). In total, the 11,457 probe-sets revealed 9,180 eQTL at LOD score > 2 (Additional file 1), 1,058 eQTL at LOD score > 3, and 160 eQTL at LOD score > 4. Only 29 had eQTL with LOD score > 5. This yielded an average of 1.5 eQTL per transcript (ranging from 1 to 6 eQTLs). The eQTL were distributed over all autosomes with some regions harboring particularly many eQTL with high significance (Figure 1). Out of the 9,180 eQTL 8,168 eQTL could be assigned to the porcine genome sequence (Ensembl Sscrofa9 database, released April 2009). In total 653 eQTL were mapped on the same chromosome as the corresponding gene itself. These eQTL are putative *cis-*eQTL.

**Table 1:** Global eQTL summary

Total # of probe-sets	Lod score threshold	Nominal *P*	Total # of eQTL	# of probe-sets with at least one eQTL	# of eQTL mapped	# of eQTL with ipsi-chromosomal location*
11,457	> 2	4.90E-03	9180	6117	8168	653
11,457	> 3	4.59E-04	1058	990	955	125
11,457	> 4	4.50E-05	160	159	139	35
11,457	> 5	3.52E-06	29	29	25	13

*, i.e. # of eQTL located on the same chromosome as the corresponding probe-set

### Integration of trait, expression, and mapping data to identify genes related to the meat quality

**Figure 2:**
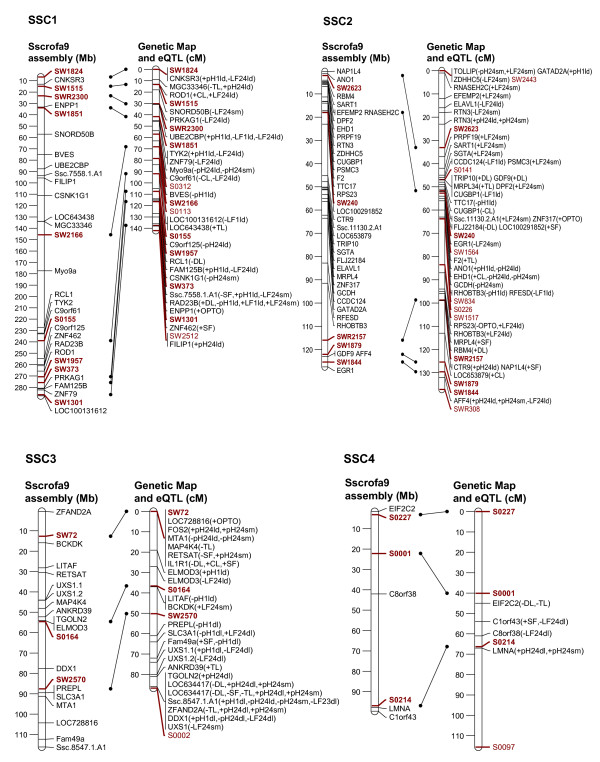
**Position of eQTL of probe-sets that show trait-correlated expression and that are located on the same chromosome (SSC1 to 4) as the corresponding genes**. Left: physical map with positions of microsatellite markers (red) and genes (black) represented by the probe-sets in Mb according to the Sscrofa9 genome sequence; Right: sex-averaged linkage maps of SSC 1 to 4 with positions of microsatellite markers and eQTL of corresponding probe-sets in cM; Both maps are linked based on the microsatellite marker positions (red and bold). Meat quality traits, with which the expression levels of probe-sets are correlated, are presented in brackets. Trait abbreviations are given at table 2.

**Figure 3:**
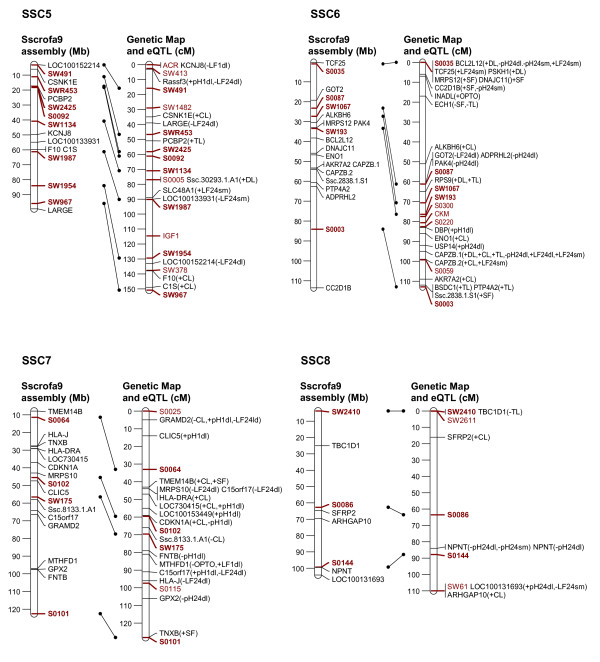
**Position of eQTL of probe-sets that show trait-correlated expression and that are located on the same chromosome (SSC5 to 8) as the corresponding genes**. Left: physical map with positions of microsatellite markers (red) and genes (black) represented by the probe-sets Mb according to the Sscrofa9 genome sequence; Right: sex-averaged linkage maps of SSC 1 to 4 with positions of microsatellite markers and eQTL of corresponding probe-sets in cM; Both maps are linked based on the microsatellite marker positions (red and bold). Meat quality traits, with which the expression levels of probe-sets are correlated, are presented in brackets. Trait abbreviations are given at table 2.

**Figure 4:**
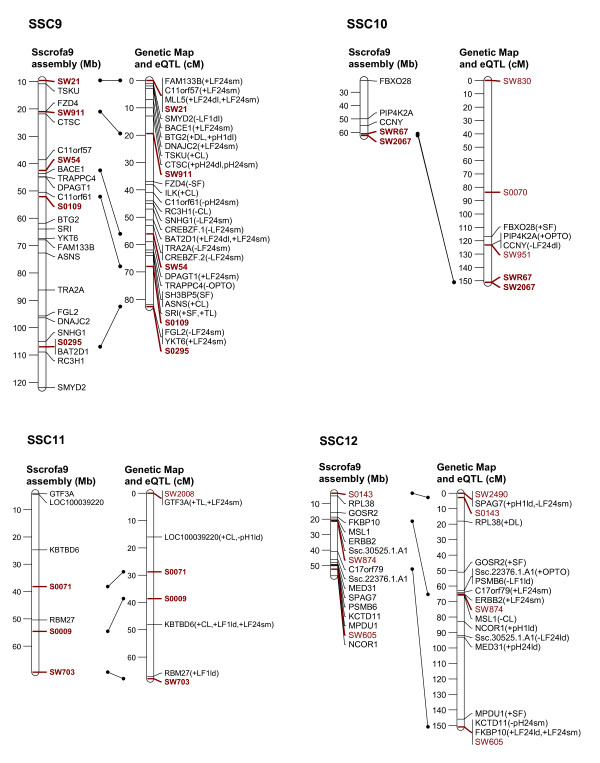
**Position of eQTL of probe-sets that show trait-correlated expression and that are located on the same chromosome (SSC9 to 12) as the corresponding genes**. Left: physical map with positions of microsatellite markers (red) and genes (black) represented by the probe-sets Mb according to the Sscrofa9 genome sequence; Right: sex-averaged linkage maps of SSC 1 to 4 with positions of microsatellite markers and eQTL of corresponding probe-sets in cM; Both maps are linked based on the microsatellite marker positions (red and bold). Meat quality traits, with which the expression levels of probe-sets are correlated, are presented in brackets. Trait abbreviations are given at table 2.

**Figure 5:**
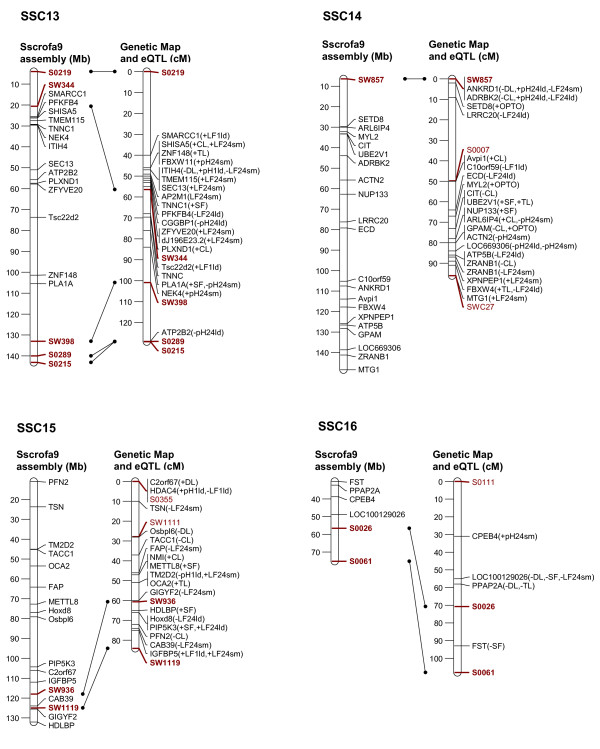
**Position of eQTL of probe-sets that show trait-correlated expression and that are located on the same chromosome (SSC12 to 16) as the corresponding genes**. Left: physical map with positions of microsatellite markers (red) and genes (black) represented by the probe-sets Mb according to the Sscrofa9 genome sequence; Right: sex-averaged linkage maps of SSC 1 to 4 with positions of microsatellite markers and eQTL of corresponding probe-sets in cM; Both maps are linked based on the microsatellite marker positions (red and bold). Meat quality traits, with which the expression levels of probe-sets are correlated, are presented in brackets. Trait abbreviations are given at table 2.

**Figure 6:**
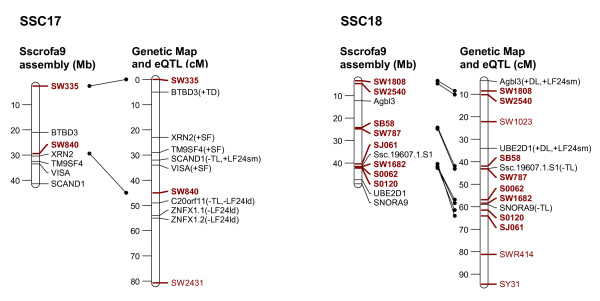
**Position of eQTL of probe-sets that show trait-correlated expression and that are located on the same chromosome (SSC17 and 18) as the corresponding genes**. Left: physical map with positions of microsatellite markers (red) and genes (black) represented by the probe-sets Mb according to the Sscrofa9 genome sequence; Right: sex-averaged linkage maps of SSC 1 to 4 with positions of microsatellite markers and eQTL of corresponding probe-sets in cM; Both maps are linked based on the microsatellite marker positions (red and bold). Meat quality traits, with which the expression levels of probe-sets are correlated, are presented in brackets. Trait abbreviations are given at table 2.

In order to link the eQTL to the genetic background of a classical phenotypic trait of interest, it is necessary to establish a relationship between the variation of that classical phenotypic trait, the expression levels of various transcripts, and the mapping position of the eQTL and the corresponding transcripts. Therefore, we selected transcripts whose expression level showed significant correlation with individual meat quality traits and with the composite traits, PC2 and PC3 (Tables 2, 3, 4) (p ≤ 0.05, corresponding to correlation coefficients ranging between |0.24-0.50|) and we focussed on those transcripts with eQTL on their own chromosome as the structurally fixed linkage group (collateral transcript and eQTL position). Out of 653 eQTL that were mapped on the chromosome of their corresponding genes 262 transcripts showed correlation with at least one meat quality trait. These are displayed in Figure 2, 3, 4, 5 and 6. The linkage map showed accurate match of microsatellites marker order with the marker arrangement in the current porcine genome sequence assembly (Sscrofa9 assembly, April 2009). A total of 73 out 115 microsatellites marker were found to align on the porcine genome sequence assembly map position by BLAST analysis. Reflecting this alignment, the position of 262 transcripts in the porcine physical map was assigned to the corresponding eQTL position in the genetic linkage maps. Most of the eQTL of transcripts whose expression level correlated with meat quality traits were found on SSC1, 2, 3, 6, 9 and 14. Table 2 summarizes the number of eQTL related to various meat quality traits.

**Table 2:** The number of eQTL of probe-sets exhibiting expression levels that are correlated with meat quality traits

		No. of eQTL with trait correlated express gene	
		
Meat quality parameters	Traits	All eQTL	eQTL located on the same chromosome as the corresponding probe-set
routine parameters			
- pH	pH1ld	425	35
	pH24ld	384	34
	pH24sm	271	28
- conductivity	LF1h loin	253	16
	LF24ld	793	50
	LF24ld	1012	64
meat texture	DL	298	26
	TL	285	28
	CL	76	40
technological meat quality parameters	QPTO	273	12
	SF	439	35

Trait abbreviations:

pH1ld pH-value in Mld at 13^th^/14^th ^rib 45 min post mortem

pH24ld pH-value in Mld at 13^th^/14^th ^rib 24 h post mortem

pH24sm pH-value in M. semimembranosus (Msm) at 24 h post mortem

LF1ld conductivity in Mld at 13^th^/14^th ^rib45 min post mortem

LF24ld conductivity in Mld at 13^th^/14^th ^rib post mortem

LF24sm conductivity in Msm at 24 h post mortem

DL Drip loss: percentage of weight loss after 48 h of Mld samples collected at 24 h post mortem

TL Thaw loss: percentage of weight loss of Mld samples frozen at -20°C.

CL Cooking loss: percentage of weight loss of Mld samples incubated in water at 75°C for 50 min

OPTO meat colour 24 h post mortem in *M. longissimus dorsi *(Mld)at 13^th^/14^th ^rib; OPTO star

SF Shear force was measured by the Instron-4310 equipment

**Table 3:** Probe-sets with expression levels that are correlated with principal component 2 and information about their eQTL.

Annotation		eQTL			individual trait correlation*
Prob_set_IDs	Gene Symbols	SSC	cM	F	traits
Ssc.25132.3.S1	RAD23B	1	130	5.11	DL, -pH1ld, LF1ld, LF24sm
Ssc.11839.1.S1	ZNF462	1	138	8.12	SF
Ssc.21636.1.A1	FILIP1	1	142	5.4	pH24ld
Ssc.21225.1.S1	RNASEH2C	2	1	5.2	LF24sm
Ssc.27245.1.S1	RTN3	2	18	10.8	-LF24sm
Ssc.5869.1.S1	SART1	2	42	8.7	LF24sm
Ssc.11130.2.A1		2	50	5.4	LF24sm
Ssc.16460.1.S1	EGR1	2	52	6.2	-LF24sm
Ssc.28435.1.A1	EHD1	2	56	8.21	CL, -pH24ld, -pH24sm, LF24sm
Ssc.6529.1.A1	AFF4	2	132	9.3	pH24ld, pH24sm, -LF24ld
Ssc.16877.1.S1	LOC728816	3	0	6.4	OPTO
Ssc.15224.3.A1	FOSL2	3	0	5.8	pH24ld, pH24sm
Ssc.1051.1.S1	TGOLN2	3	86	5.9	pH24ld
Ssc.15224.2.S1	FOSL2	3	86	6.2	-DL, pH24ld, pH24sm
Ssc.15224.1.S1	FOSL2	3	86	6.3	-DL, -SF, -TL, pH24ld, pH24sm
Ssc.24035.1.S1	EIF2C2	4	45	7.4	-DL, -TL
Ssc.5112.2.S1	LMNA	4	67	9.54	pH24ld, pH24sm
Ssc.3935.1.S1	SLC48A1	5	89	5.2	LF24sm
Ssc.26780.1.S1	BCL2L12	6	0	6.9	DL, -pH24ld, -pH24sm, LF24sm,
Ssc.1545.1.A1	TCF25	6	0	5.7	LF24sm
Ssc.20488.1.A1	RPS9	6	65	8.4	DL, TL
Ssc.14436.1.S1	USP14	6	92	5.4	pH24ld
Ssc.10297.3.S1	CAPZB	6	95	6.2	DL, CL, TL, -pH24ld, LF24ld, LF24sm
Ssc.10297.1.S1	CAPZB	6	99	6	CL, LF24sm
Ssc.21139.2.S1	CLIC5	7	14	6	pH1ld
Ssc.10148.1.S1	MTHFD1	7	81	16.1	-OPTO, LF1ld
Ssc.22107.1.A1	LOC100131693	8	110	5.8	pH24ld, LF24sm
Ssc.16983.1.S1	TRAPPC4	9	62	7.7	-OPTO
Ssc.22376.1.A1		12	63	5.5	OPTO
Ssc.4217.1.S1	ITIH4	13	47	7.3	-DL, pH1ld, -LF24sm
Ssc.20319.1.S1	TMEM115	13	47	5.2	LF24sm,
Ssc.31206.3.S1	CGGBP1	13	52	5.9	-pH24ld,
Ssc.10429.1.S1	ANKRD1	14	0	6	-DL, pH24ld, -LF24sm
Ssc.10911.1.A1	ADRBK2	14	0	6	-CL, pH24ld, -LF24ld
Ssc.18050.1.S1	ZRANB1	14	87	5.6	-LF24sm
Ssc.11397.1.A1	MTG1	14	96	6.7	LF24sm
Ssc.6058.1.S1	LOC100129026	16	55	4.9	-DL, -SF, -LF24sm
Ssc.18640.3.S1	UBE2D1	18	34	4.9	DL, LF24sm

"*"the probe-sets listed here represent a subset of probe-sets correlated with individual meat quality traits as indicated in this column naming the traits with which the corresponding probe-sets show correlated expression in addition to the correlated expression with PC2; "-" indicates negative correlation

Trait abbreviations are given at table 2.

**Table 4:** Probe-sets with expression levels that are correlated with principal component 3 and information about their eQTL

Annotation		eQTL			individual trait correlation*
Prob_set_IDs	Gene Symbols	SSC	cM	F	traits
Ssc.16910.1.S1	ROD1	1	13	5.92	CL, LF24ld
Ssc.26338.1.S1	PRKAG1	1	30	6.69	-LF24ld
Ssc.29222.1.S1	UBE2CBP	1	31	5.72	pH1ld, -LF1ld, -LF24ld
Ssc.3853.1.S1	C9orf61	1	54	7.98	-DL, pH1ld, -LF24sm
Ssc.20392.1.S1	BVES	1	63	22.1	-pH1ld
Ssc.7558.1.A1		1	127	7.96	-SF, pH1ld, -LF24ld
Ssc.25132.3.S1	RAD23B	1	130	5.11	DL, -pH1ld, LF1ld, LF24sm
Ssc.16460.1.S1	EGR1	2	52	6.24	-LF24sm
Ssc.29893.1.A1	RHOBTB3	2	94	7.37	LF24ld
Ssc.14340.3.S1	LITAF	3	37	6.69	-pH1ld
Ssc.7947.1.A1	PREPL	3	61	5.13	-pH1ld
Ssc.2441.2.A1	SLC3A1	3	64	6.08	-pH1ld, LF24ld
Ssc.24213.2.S1	UXS1	3	74	5.71	LF24ld, LF24sm
Ssc.5415.1.S1	DDX1	3	86	4.96	pH1ld, -pH24ld, -LF24ld
Ssc.8547.1.A1		3	86	7.11	pH1ld, LF24sm
Ssc.19258.1.S1	KCNJ8	5	0	5.98	-LF1ld
Ssc.5769.1.S1	Rassf3	5	3	7.01	pH1ld, -LF24ld
Ssc.3935.1.S1	SLC48A1	5	89	5.17	LF24sm
Ssc.10297.3.S1	CAPZB	6	95	6.17	DL, CL, TL, -pH24ld, LF24ld, LF24sm
Ssc.10297.1.S1	CAPZB	6	99	6	CL, LF24sm
Ssc.2152.2.S1	BSDC1	6	112	4.92	TL
Ssc.25358.1.S1	GRAMD2	7	5	5.41	-CL, pH1ld, -LF24ld
Ssc.21139.2.S1	CLIC5	7	14	6	pH1ld
Ssc.17920.2.S1	C15orf17	7	44	7.21	LF24sm
Ssc.30959.1.A1	LOC100153449	7	55	6.11	pH1ld
Ssc.17920.1.A1	C15orf17	7	91	7.56	-LF24sm
Ssc.8611.2.S1	MLL5	9	0	6.06	LF24ld, LF24sm
Ssc.28945.3.S1	FAM133B	9	0	5.15	LF24sm
Ssc.23527.1.A1	C11orf57	9	0	7.79	LF24sm
Ssc.8415.1.A1	BACE1	9	2	5.28	LF24sm
Ssc.16454.1.S1	DNAJC2	9	7	5.51	LF24sm
Ssc.6155.1.S1	CTSC	9	13	8.95	pH24ld, pH24sm
Ssc.28609.1.S1	SNHG1	9	45	5.21	-LF24sm
Ssc.25107.1.S1	BAT2D1	9	51	6.56	LF24ld, LF24sm
Ssc.1537.1.S2	YKT6	9	82	5.23	LF24sm
Ssc.8563.2.S1	RBM27	11	67	5.11	LF24ld
Ssc.30812.1.S1	SPAG7	12	0	5.02	pH1ld, LF24sm
Ssc.6130.1.S1	FBXW11	13	46	8.08	pH24sm
Ssc.4217.1.S1	ITIH4	13	47	7.31	-DL, pH1ld, -LF24sm
Ssc.6767.1.S1	dJ196E23.2	13	53	5.05	LF24sm
Ssc.7384.1.A1	ZFYVE20	13	53	7.69	LF24sm
Ssc.30107.1.S1	C10orf59	14	50	6.38	-LF1ld
Ssc.11118.1.S1	ATP5B	14	86	5.44	-LF24ld
Ssc.24012.1.S1	TSN	15	10	5.26	-LF24ld
Ssc.9483.1.A1	Osbpl6	15	28	7.28	-LF24sm
Ssc.13904.1.S1	TM2D2	15	51	6.73	-pH1ld, LF24sm
Ssc.13358.1.A1	Agbl3	18	4	5.67	DL,LF24sm

"*"the probe-sets listed here represent a subset of probe-sets correlated with individual meat quality traits as indicated in this column naming the traits with which the corresponding probe-sets show correlated expression in addition to the correlated expression with PC3; "-" indicates negative correlation

Trait abbreviations are given at table 2.

Most of these meat quality parameters were correlated or dependent on each other. Such correlations were reported by many studies [11-13]. In a previous study we used principle components (PCs) to reduce the multi dimensional data sets into lower dimensions [13]. The principal component (PC2) is a meat quality vector and has high loadings for pH24ld, pH24st and meat color which are inversely correlated with drip loss. The principal component (PC3) is also a meat quality vector with large positive contribution of conductivity (LF1ld, LF24ld, LF24sm) and negative contribution of pH1ld. In order to scale down the list of 262 putative *cis-*eQTL for meat quality, transcript levels of genes correlated with the two PCs with high loadings of meat quality traits were considered. The level of expression of 38 and 47 probe-sets, respectively, were correlated with the new composite traits that were generated by principal component analysis PC2 and PC3 (Tables 3 and 4). In order to unravel the composite traits, the correlation of these 38 and 47 transcripts with each single meat quality trait contributing to the PC was screened and traits with significant correlations with the respective genes are given in tables 3 and 4. Thus the probe-sets represent a subset of probe-sets correlated with individual meat quality traits. As previously shown, analyses of enrichment of functional annotation groups as defined in the Gene Ontology (GO), the Kyoto Encyclopedia of Genes and Genomes (KEGG) databases, or in the Ingenuity Pathways Analysis library, highlight ubiquitination, phosphorylation, mitochondrion dysfunction, actin-, integrin-, PDGF-, EGF-, VEGF-, and Ca-signalling pathways [13]. Most of the eQTL related to PC2 were found on chromosomes 2, 3, and 6; for PC3 on chromosomes 1, 3, 7, and 9.

## Discussion

Complex traits are genetically controlled by many loci. The identification of candidate genes for these traits is widely based on the principle of 'collecting evidences' in order to prioritize genes for further analysis from the huge lists of functional and positional candidate genes. The detection of eQTL provides information about the positional aspects of gene expression regulation. Together with the growing knowledge of genome sequences and gene annotation, this gives insight into the architecture of regulatory networks. In order to relate eQTL analysis to the genetic background of any complex trait, either a positional link to QTL for the complex trait of interest (pQTL) and/or a functional link to the trait expression is required. Here we report on the detection of eQTL for some 11,000 transcripts found in porcine skeletal muscle by microarray analysis. We demonstrate that the global microarray eQTL analysis can serve for narrowing down the candidate genes for quantitative traits related to meat quality when it is integrated with the analysis of the correlation of expression levels with the traits of interest.

The genetic architecture of transcript level variation in the porcine F2 resource population was highly variable and complex. eQTL were detected for 53% of the 11,457 probe-sets, with many of them exhibiting more than one eQTL summing up to more than 9000 eQTL in total (Table 1). The expression levels of the majority of the transcripts showed quantitative variation. So the transcript levels are probably quantitatively controlled. The link between the linkage maps and porcine assembly sequence enable discriminating *cis *or *trans*-eQTL. All 23,934 probe-sets sequence represented on the porcine microarray and the microsatellites sequences were BLASTed against the Ensembl porcine BAC sequence (Sscrofa9, April 2009). Our microsatellite order showed high accuracy compared to the Ensembl porcine BAC sequence.

Correlations between transcript abundance and meat quality, combined with genetic positional information of eQTL allowed us to prioritize a small number of candidate genes. We considered transcripts, which exhibited expression levels correlated with meat quality traits, and which had eQTL on the same chromosome as the transcripts itself.

Our previous studies showed that *cis *regulation is a stable characteristic of individual transcripts. Consequently, a global microarray eQTL analysis of a limited number of samples can be used for exploring functional and regulatory gene networks and scanning for *cis-*eQTL. In particular, the assignment of eQTL to chromosomes is reliable; though some *cis-*eQTL change their position, they were consistently assigned to the same chromosome when comparing analyses based on 74 microarrays or 276 real time RT-PCRs [7]. Based on this observation, we decided to highlight only eQTL which were located on the gene itself or the same chromosome. Moreover, this relaxed measure was chosen in order to avoid the exclusion of any true *cis*-acting eQTL that are not precisely mapped, because of the limited resolution of the genetic map. The observation that *cis-*eQTL were more consistently detected than *trans-*eQTL was also made by comparing the results from different studies of eQTL with different numbers of animals and different tissue types [14-17].

Expression QTL mapping, with its potential to categorize *cis *and *trans-*effects, provides the mean to discriminate between "effect" and "cause" with respect to trait-associated differential expression. Though a relaxed window of *cis*-eQTLs was used, a low proportion of 10% of putative *cis*-eQTL was found in this study, compared to other previous studies [18,19]. *Cis*-regulated genes are of interest, because the underlying genes are expected to harbour genetic variants that influence their own expression level, which may also influence the physiological traits of interest, if transcript abundance is correlated with the target phenotype [17]. This provides a function-related evidence of the candidacy of a particular gene.

The usefulness of eQTL for identification of quantitative trait genes was demonstrated [3,5,20,21]. The causal link between sequence variation, gene expression, and phenotype arises because the polymorphism might be responsible for both a *cis-*eQTL and the QTL contributing to a quantitative phenotype, so called pQTL. QTL for traits related to meat quality were previously mapped in the population used here [12,22]. The matching of pQTL, eQTL and the localization of the corresponding genes with trait-correlated expression provides positional and functional evidence for the potential role of the respective genes and strongly promote them as candidate genes. Mapping of eQTL enables displaying regulatory networks and localizing genomic variation affecting the mRNA expression of a gene either within the genes itself (*cis*) or distant from the gene (*trans*). The key advantage of eQTL mapping is that it connects variation at the level of RNA expression to variation at the level of DNA. Only the latter provides versatile tools for breeding whereas the first reveals information on the biology of a trait and directs to new candidate genes. Moreover, integration of (1) information on QTL for a trait of interest in breeding (pheneQTL = pQTL) with analysis of (2) trait correlated expression and with (3) mapping of expression QTL (eQTL) for the corresponding trait-dependent-regulated genes facilitates the identification of genes and pathways with cumulative evidence of their involvement in the biology of the traits of interest and enable to built priority lists of candidate genes [23]. However, there are also some issues that limit of the use of the genetical genomics approach, in particular the resolution of the genetic maps that is depending on the number of markers and animals used and the structure of the population used and artifacts caused by the limited sensitivity and specificity of microarray experiments [24].

In a previous study we used the principle components with high loadings of meat quality traits to identify functional networks of genes and to gain knowledge of biological and physiological processes taking place during the conversion of muscle to meat [13]. Here in this study, we combined principle component analysis of traits related to meat quality with eQTL to scale down the list of candidate genes for the traits.

## Conclusion

Holistic expression profiling was integrated with QTL analyses for meat quality traits. This is, to our knowledge, the first report of a comprehensive scan for *cis-*eQTL associated with meat quality traits in the pig. Correlations between transcript abundance and meat quality traits, combined with genetic positional information of eQTL allowed us to prioritise candidate genes for further study. Accordingly, a list of candidate genes for meat quality was set up. The further identification of the causative polymorphisms and the determination of their functional role are even more challenging, since there are several different molecular mechanisms through which mRNA levels in cells can be regulated.

## Methods

### Animals and tissue collection

This study was based on trait measurements, genotyping procedures, expression profiling, and linkage analysis performed in the three-generation resource family (DuPi) founded by crossbreeding Duroc and Pietrain and described in detail by Liu *et al. *[12,22]. All animals were free of the mutation at the ryanodine receptor locus, RYR1, which is responsible for malignant hyperthermia syndrome. A total of 572 F2 animals comprising 31 full-sib families were used for recording of meat performance traits and construction of a linkage map comprising genotypes of 115 microsatellite markers. Expression profiling and eQTL analysis were done with 74 F2 animals of this resource population that represented a subset of the population covering 25 full-sib families derived from all five F1 boars of the population and 18 out of 27 F1 sows. The experimental research on animals was done according to the German Animal Welfare Act and approved by the animal welfare board of the Leibniz Institute of Farm Animal Biology, FBN Dummerstorf.

### Traits and phenotypes

Phenotypic data of F2 animals were collected following the guidelines of the German performance test (ZDS 2003) [25]. After slaughter technological parameters of meat quality, i.e. pH-value, conductivity and colour, were measured by using Star-series equipment (Rudolf Matthaeus Company, Germany). Measures of pH and conductivity were at 45 min post mortem (pH1) and 24 h post mortem (pH24), respectively; both in *M. longissimus dorsi *between 13^th^/14^th ^rib (pH1ld, pH24ld, LF1ld, LF24ld) and in the ham (*M. semimembranosus*) (pH24sm, LF24sm), respectively. Muscle colour was measured at 24 h post mortem by Opto-Star. Drip loss was scored based on a bag-method with a size-standardized sample from the *M. longissimus dorsi *collected at 24 hours post mortem that was weighted, suspended in a plastic bag, held at 4°C for 48 h, and thereafter re-weighed [26,27]. To obtain cooking loss, a loin cube was taken from the *M. longissimus dorsi*, weighed, placed in a polyethylene bag and incubated in water at 75°C for 50 minutes. The bag was then immersed in flowing water at room temperature for 30 minutes and the solid portion was re-weighed. Thawing loss was determined similarly after at least 24 h of freezing at -20°C. Drip loss, cooking loss, and thawing loss were calculated as a percentage of weight loss based on the start weight of a sample. Shear force was measured by the Instron-4310 equipment and the average values of four replicates were used for analyses. The procedure 'Factor' of the SAS software package (SAS version 9.1 SAS Institute, Cary, NC) was used to derive four principle components (PCs) based on 19 traits related to muscle and carcass properties of 572 DUPI animals; i.e. the dimensionality of the data was reduced from the original 19 traits to 4 principal components (PCs), where each PC is a linear combination of all traits without a significant loss of information. PC1 and PC4 represent variation in carcass traits, whereas PC2 and PC3 represent aspects of meat quality, consequently PC2 and PC3 were used to correlate with expression profiles. The principal component analysis of meat quality traits is discussed in Ponsuksili et al., 2009 [13].

### Whole genome expression profiling

Immediately *post mortem *tissue samples were collected and snap frozen that were taken between the 13th and 14th rib from the center of *M. longissimus dorsi *of 74 animals. Total RNA was isolated using TRI Reagent (Sigma, Taufkirchen, Germany) according to the manufacturer's protocol. After DNaseI treatment the RNA was cleaned up using the RNeasy Kit (Qiagen, Hilden, Germany). The quantity of RNA was established using the NanoDrop ND-1000 spectrophotometer (Peqlab, Erlangen, Germany) and the integrity was checked by running 1 μg of RNA on 1% agarose gel. In addition absence of DNA contamination was checked using the RNA as a template in standard PCR amplifying fragments of *RPL32 *and *HPRT1*. Muscle expression pattern were assessed using 74 Porcine Genome Array which contains 24,123 probe-sets that interrogate 20,689 transcripts that were assigned to known genes [6]. Preparation of target products, hybridization and scanning using the GeneChip scanner 3000 were performed according to Affymetrix protocols using 5 μg of total RNA to prepare antisense biotinylated RNA. The quality of hybridization was assessed in all samples following the manufacturer's recommendations. Data were analysed with Affymetrix GCOS 1.1.1 software using global scaling to a target signal of 500. Data were then imported into Arrays Assist software (Stratagene Europe, Amsterdam, The Netherlands) for subsequent analysis. The data were processed with MAS5.0 to generate cell intensity files (using default settings with detection P-values of < 0.04 for 'present', ≥0.04 and ≤ 0.06 for 'marginal', < 0.06 for 'absent'; only 'present' calls were used). Quantitative expression levels of the present transcripts were estimated using PLIER (Probe Logarithmic Intensity Error) for normalization. The microarray data related to all samples have been deposited in the Gene Expression Omnibus (GEO, [28]) public repository (GEO accession number: GSE10204).

### Correlation between phenotype and gene expression

Phenotypic data, i.e. expression levels and meat quality data as well as PC traits, were adjusted for systematic effects by analysis of variance performed with the procedure 'Mixed' of the SAS software package (SAS System for Windows, Release 9.02) before analysing their correlation and performing eQTL analysis. Full-sib family and sex were used as fixed effects, carcass weight as a covariate and slaughter date as random effect. Based on observations from 74 animals Pearson correlation coefficients were calculated between the residuals of the log2 transformed expression intensities of all 11,453 probes and each individual meat quality trait as well as between the expression levels and both composite traits, PC2 and PC3. For each pair of transcript level and phenotype, Pearson correlation together with the P-value was computed (significance threshold p ≤ 0.05). The correlation coefficients at p ≤ 0.05 ranged between |0.24-0.50|. The observed false discovery rates were 0.08-0.34 which is reasonable for a microarray study, in particular, considering the relatively relaxed p-value (p ≤ 0.05).

### eQTL analysis

In order to map eQTL adjusted expression values of 11,457 probe-sets were regressed onto the additive and dominance coefficients in intervals of 1 cM using the F2 option of QTL express [29]. Chromosome-wide significance levels were estimated by permutation tests using 5000 permutations [30]. The 5% chromosome-wide threshold corresponds approximately to the suggestive linkage threshold proposed by Lander & Kruglyak (1995) [10]. Average significance thresholds were 4.92 corresponding to LOD = 2.0 with nominal p ≤ 10^-3^. Annotations and localization of probe-sets was based on assembly Sscrofa9 (April 2009) [6]. In order to link the genetic map to Ensembl Sscrofa9, all 115 microsatellites sequences, which were used in the linkage map, were BLASTed against the Ensembl porcine BAC sequence (Sscrofa9, April 2009). Only 73 microsatellites sequences could be localized on Sscrofa 9 as shown on Figure 2, 3, 4, 5, 6.

## Authors' contributions

SP analyzed the microarray data and drafted the manuscript; EM, MS, and KS aided in data analysis and helped in drafting the manuscript; KW conceived and designed the study, contributed to data interpretation and helped in drafting the manuscript. All authors read and approved the final manuscript.

## Supplementary Material

Additional file 1 **eQTL detected for probe-sets representing transcripts that are expressed in porcine *M. longissimus dorsi***. All 9,180 eQTL (at LOD score > 2) estimated for probe-sets of the Affymetrix Porcine Genome Array, their position in cM on linkage groups, corresponding gene names and chromosomal positions according to the current porcine genome sequence assembly (Sscrofa9 assembly, April 2009) [6] Click here for file
